# Supplementary material for: Prognostic Impact of Sarcopenia in Patients with Advanced Prostate Carcinoma: A Systematic Review
Source: J Clin Med. 2022 Dec 21;12(1):57. doi: 10.3390/jcm12010057 (PMC9821501; doi:10.3390/jcm12010057)
Supplement: Supplementary file 1 [file jcm-12-00057-s001.zip › Supplementary_Figures.pdf]

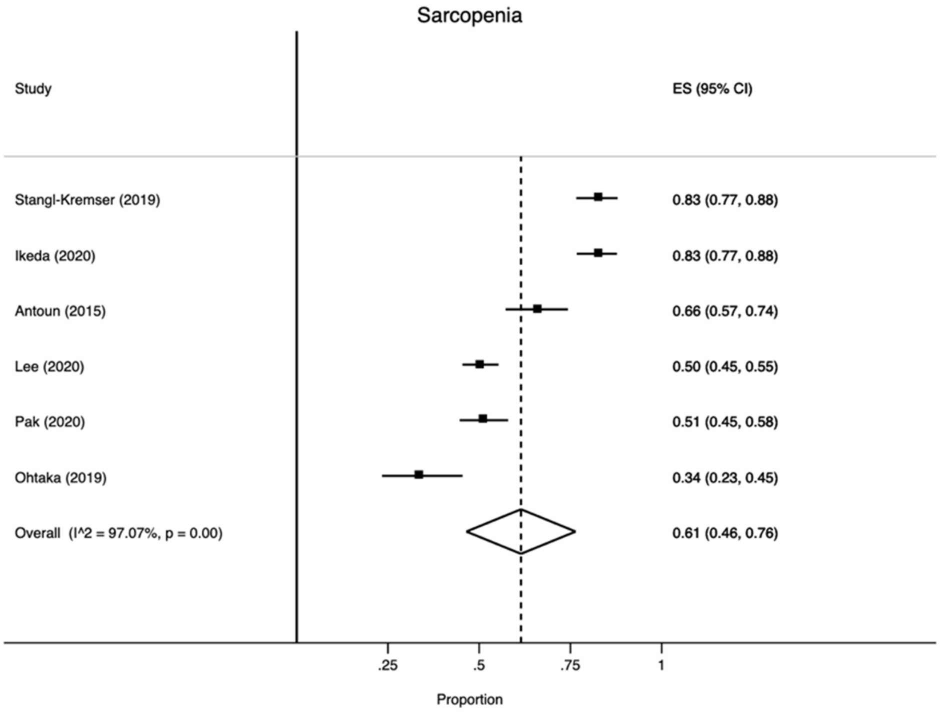

Figure S1. Forest plot for sarcopenia prevalence.

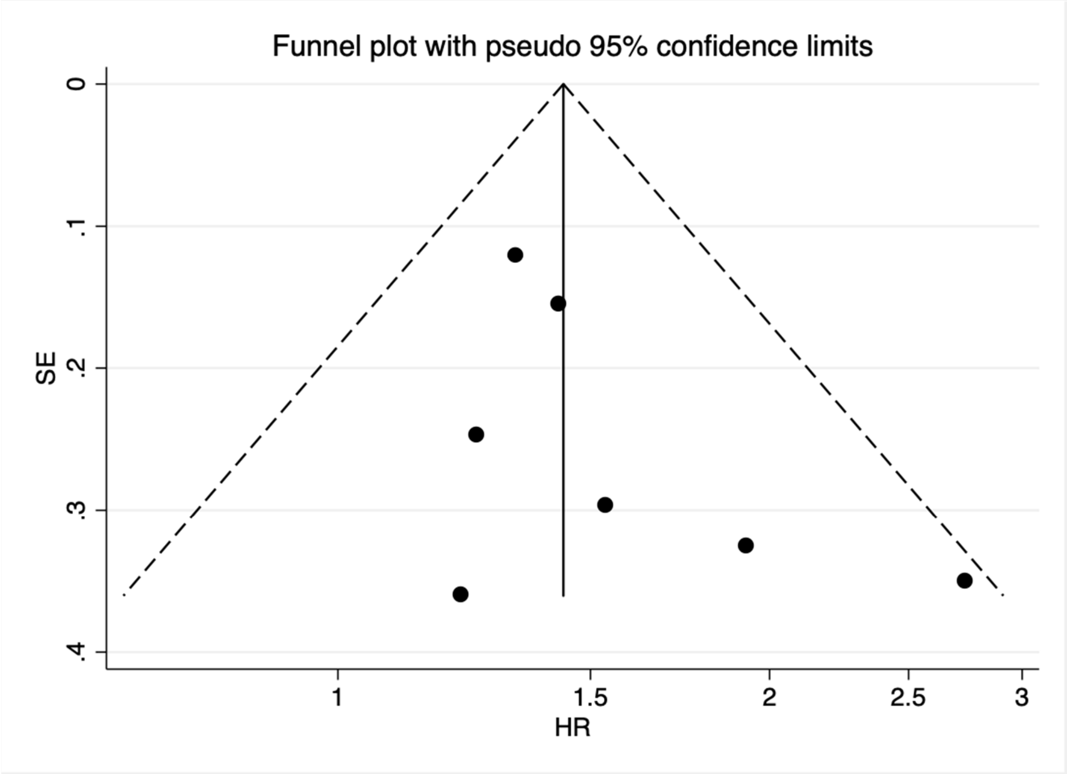

Figure S2. Funnel plot—publication bias: Univariate analysis of overall survival.

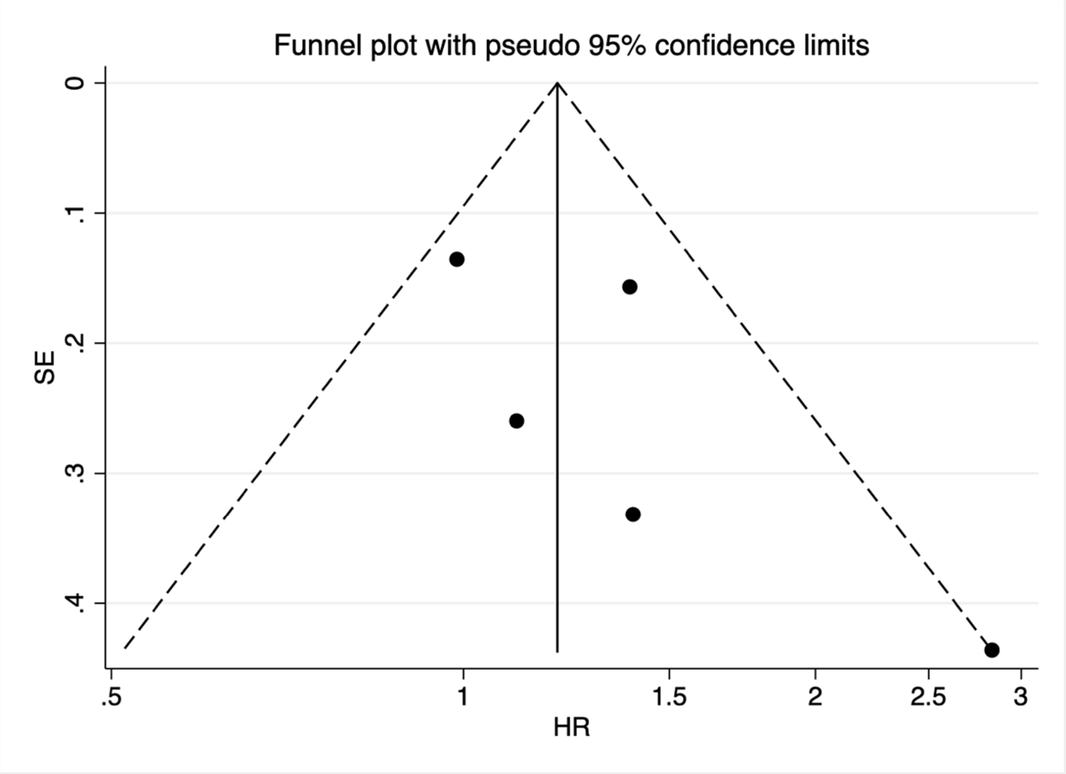

Figure S3. Funnel plot—publication bias: multivariate analysis of overall survival.

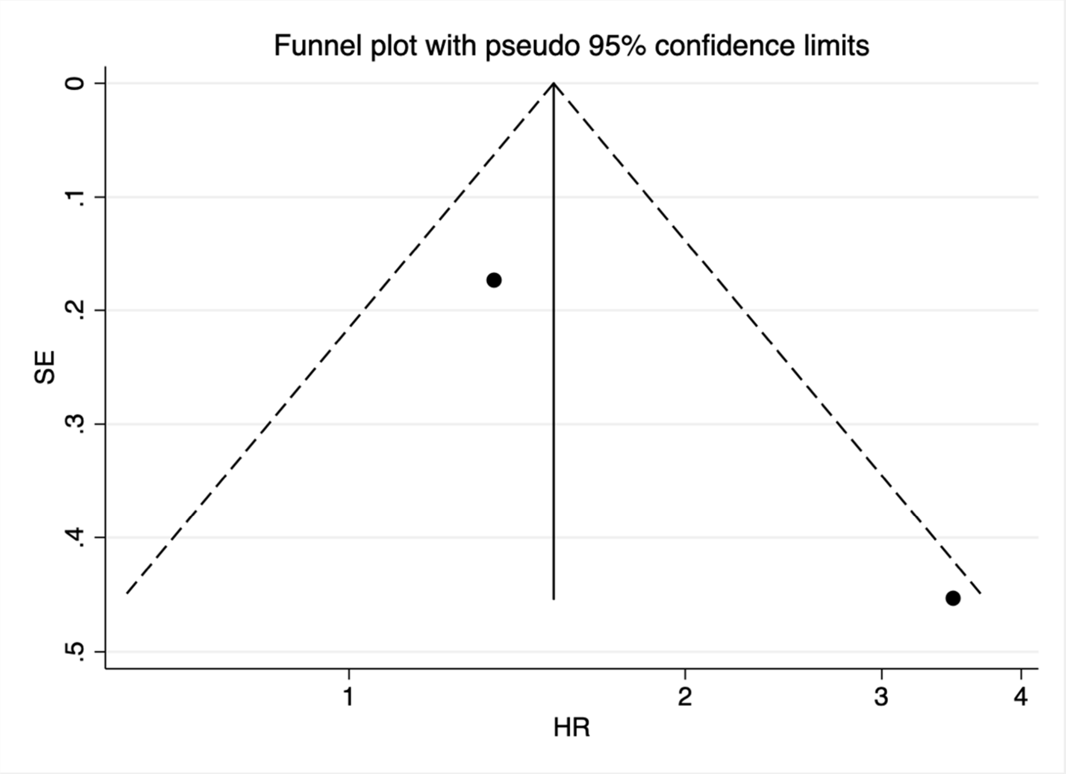

Figure S4. Funnel plot—publication bias: univariate analysis of cancer-specific survival.

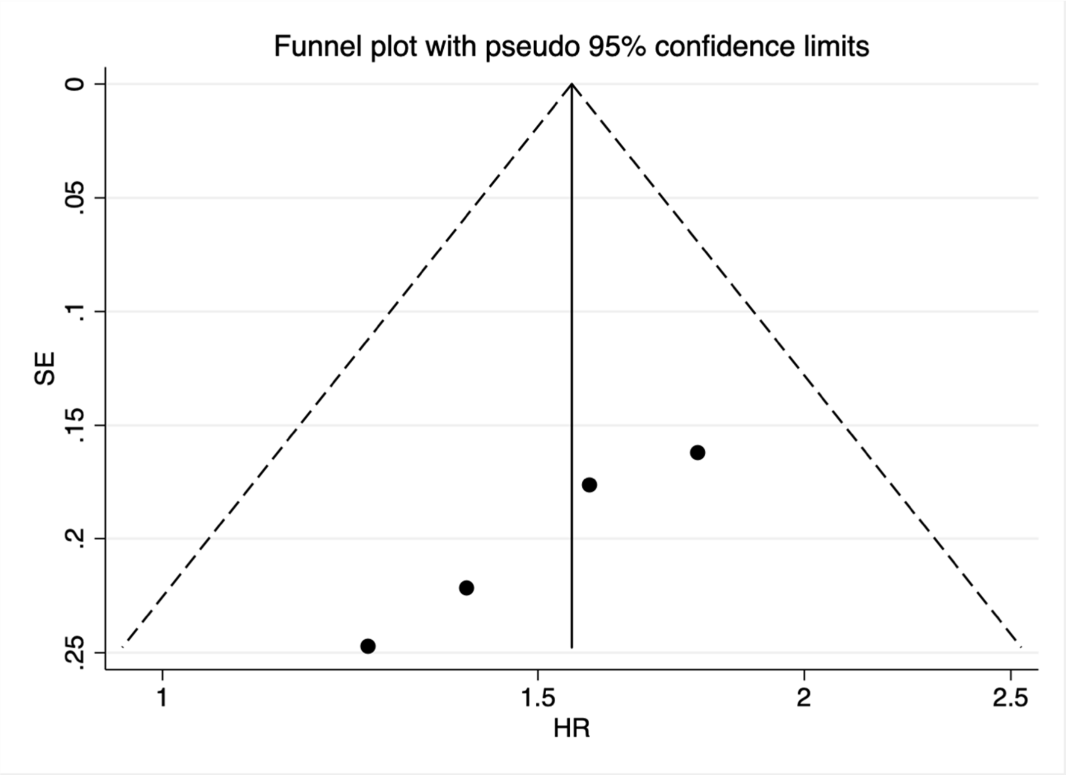

Figure S5. Funnel plot—publication bias: univariate analysis of disease-free survival.

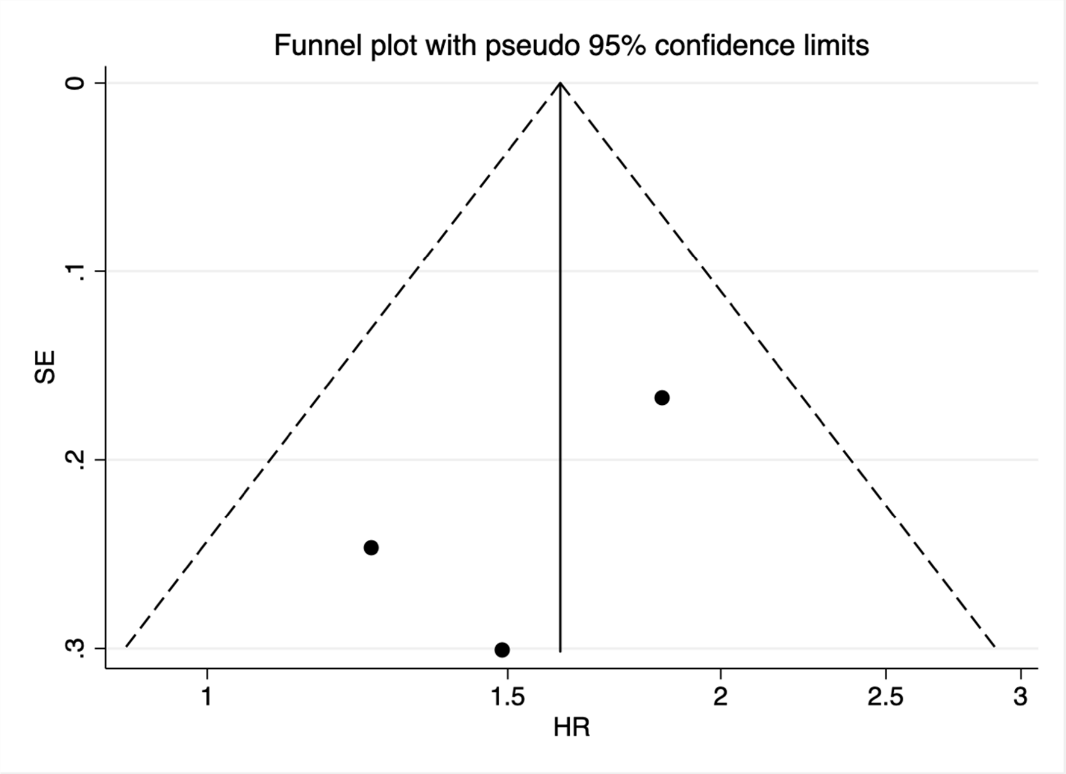

Figure S6. Funnel plot—publication bias: multivariate analysis of disease-free survival.
