# Supplementary material for: Prognostic Impact of Sarcopenia in Patients with Advanced Prostate Carcinoma: A Systematic Review
Source: J Clin Med. 2022 Dec 21;12(1):57. doi: 10.3390/jcm12010057 (PMC9821501; doi:10.3390/jcm12010057)
Supplement: Supplementary file 1 [file jcm-12-00057-s001.zip › Supplementary_Tables.pdf]

Table S1. Search strategy.

Medline
1. exp Prostatic Neoplasms/
2. (Prostat* adj5 (Neo?plasm$ or cancer or tumo?r or carcinom* or adenoma* or adenocarcin* or mass or masses or cyst* or oncolog* or sarcom* or malignan$*)).tw.
3. prostat*.hw. and exp Neoplasms/
4. 1 or 2 or 3
5. "Aged, 80 and over"/ or Aged/
6. Frail Elderly/
7. ((("60" or "65" or 60-65) adj (elder* or adult* or aged or age or old or older or over or year*)) or elder* or adult*).mp.
8. 5 or 6 or 7
9. 4 and 8
10. Sarcopenia/
11. (muscle* adj4 (deplet* or loss or wasting or reduction or skeletal or attenuation or strength or fatigue or atrophy)).ti,ab.
12. sarcopen*.ti,ab,kw.
13. exp Cachexia/
14. cache*.ti,ab.
15. 10 or 11 or 12 or 13 or 14
16. 9 and 15
17. limit 16 to (english language or spanish)
18. limit 17 to humans
**Embase**
1. 'prostate tumor'/exp
2. (prostat* NEAR/5 (neo?plasm* OR cancer OR tumo?r OR carcinom* OR adenoma* OR adenocarcin* OR mass OR masses OR cyst* OR oncolog* OR sarcom* OR malignan$*)):ti,ab,de
3. prostat*:ti,ab,de AND 'neoplasm'/exp
4. #1 OR #2 OR #3
5. 'aged'/exp
6. 'very elderly'/exp
7. 'frail elderly'/exp
8. ((('60' OR '65' OR '60-65') NEAR/1 (elder* OR adult* OR aged OR age OR old OR older OR over OR year*)):ti,ab,de) OR elder*:ti,ab,de OR adult*:ti,ab,de
9. #5 OR #6 OR #7 OR #8
10. 'sarcopenia'/exp
11. (muscle* NEAR/4 (deplet* OR loss OR wasting OR reduction OR skeletal OR attenuation OR strength OR fatigue OR atrophy)):ti,ab
12. sarcopen*:ti,ab,de
13. 'cachexia'/exp
14. cache*:ti,ab
15. #10 OR #11 OR #12 OR #13 OR #14
16. #4 AND #9 AND #15
17. #4 AND #9 AND #15 AND ([english]/lim OR [spanish]/lim)
18. #4 AND #9 AND #15 AND ([english]/lim OR [spanish]/lim) AND [humans]/lim
**WOS**
1. TS= (Prostat* NEAR/5 (Neo?plasm* or cancer or tumo?r or carcinom* or adenoma* or adenocarcin* or mass or masses or cyst* or oncolog* or sarcom* or malignan*))
2. TS= ((("60" or "65" or 60-65) NEAR/1 (elder* or adult* or aged or age or old or older or over or year*)) or elder* or adult*)
3. TS= sarcopen*
4. TS= (muscle* NEAR/4 (deplet* or loss or wasting or reduction or skeletal or attenuation or strength or fatigue or atrophy))
5. TS= cache*
6. #5 OR #4 OR #3
7. #6 AND #2 AND #1

Table S2. Results of meta-analysis, subgroup analysis and publication bias.

Variable	Model	K	N	HR	95% CI	I^2 (%)	Test Edger [IC95%]
**OVERALL SURVIVAL**							
Univariate	Fixed	7	1081	1.44	1.23, 1.67	0	0.21
Multivariate	Fixed	5	831	1.20	1.01, 1.44	43	0.20
Sensitivity analysis / Study omitted							
Antoun 2015	Random	4	711	1.33	0.95, 1.86	57	
Ikeda 2020	Random	4	634	1.27	0.93, 1.73	56	
Lee 2020	Fixed	4	624	1.40	1.11, 1.77	12	
Ohtaka 2019	Fixed	4	754	1.16	0.97, 1.39	0	
Pak 2020	Fixed	4	601	1.12	0.90, 1.39	48	
**CANCER-SPECIFIC SURVIVAL**							
Univariate	Random	2	479	1.98	0.80, 4.40	74	NA
**PROGRESSION-FREE SURVIVAL**							
Univariate	Fixed	4	818	1.56	1.29, 1.88	0	0.02
Multivariate	Fixed	3	588	1.61	1.26, 2.06	0	0.43

Note: Fixed: fixed effect; HR: hazard ratio; NA: No apply; Random: Random effect.

Table S3. Results of meta-analysis, subgroup analysis and publication bias.

Variable	Model	K	N	HR	95% CI	I^2 (%)	Subgroup Differences (p-Value)	Test Edger [IC95%]
**OVERALL SURVIVAL**								
**Univariate**	**Fixed**	**7**	**1081**	**1.44**	**1.23, 1.67**	**0**		**0.21**
Disease stage								
CRCPa	Fixed	6	884	1.41	1.20, 1.65	0	0.35	
Hormone sensitive PCa	Fixed	1	197	1.93	1.02, 3.65	NA		
Treatment								
ADT	Fixed	6	895	1.43	1.22, 1.68	3	0.81	
Chemotherapy	Fixed	1	186	1.54	0.86, 2.76	NA		
**Multivariate**	**Fixed**	**5**	**831**	**1.20**	**1.01, 1.44**	**43**		**0.20**

Disease stage								
CRCPa	Random	4	634	1.19	0.99, 1.43	56	0.64	
Hormone sensitive PCa	Fixed	1	197	1.40	0.73, 2.68	NA		
Treatment								
ADT	Fixed	5	831	1.20	1.01, 1.44	43	NA	
Sensitivity analysis / Study omitted								
Antoun 2015	Random	4	711	1.33	0.95, 1.86	57		
Ikeda 2020	Random	4	634	1.27	0.93, 1.73	56		
Lee 2020	Fixed	4	624	1.40	1.11, 1.77	12		
Ohtaka 2019	Fixed	4	754	1.16	0.97, 1.39	0		
Pak 2020	Fixed	4	601	1.12	0.90, 1.39	48		
**CANCER-SPECIFIC SURVIVAL**								
**Univariate**	**Random**	**2**	**479**	**1.98**	**0.80, 4.90**	**74**		**NA**
Disease stage								
CRCPa	Fixed	1	282	1.35	0.96, 1.90	NA	0.05	
Hormone sensitive PCa	Fixed	1	197	3.48	1.43, 8.47	NA		
Treatment								
ADT	Random	2	479	1.98	0.80, 4.90	74	NA	
**Multivariate**	**Fixed**	**1**	**197**	**2.18**	**1.07, 4.44**	**NA**		**NA**
Disease stage								
Hormone sensitive PCa	Fixed	1	197	2.18	1.07, 4.44	NA	NA	
Treatment								
ADT	Fixed	1	197	2.18	1.07, 4.44	NA	NA	
**PROGRESSION-FREE SURVIVAL**								
**Univariate**	**Fixed**	**4**	**818**	**1.56**	**1.29, 1.88**	**0**		**0.02**
Disease stage								
CRCPa	Fixed	4	818	1.56	1.29, 1.88	0	NA	
Treatment								

ADT	Fixed	3	632	1.60	1.29, 1.97	0	0,57	
Chemotherapy	Fixed	1	186	1.39	0.90, 2.15	NA		
**Multivariate**	**Fixed**	**3**	**588**	**1.61**	**1.26, 2.06**	**0**		**0.43**
Disease stage								
CRCPa	Fixed	3	588	1.61	1.26, 2.06	0	NA	
Treatment								
ADT	Fixed	3	588	1.61	1.26, 2.06	0	NA	

Note: ADT: androgen deprivation therapy; CRPCa: Castration-resistant prostate cancer; Fixed: fixed effect; HR: hazard ratio; NA: No apply; Random: Random effect; PCa: Prostate cancer.
